# Phylogenetic Insights from a Novel *Rehubryum* Species Challenge Generic Boundaries in Orthotrichaceae

**DOI:** 10.3390/plants14152373

**Published:** 2025-08-01

**Authors:** Nikolay Matanov, Francisco Lara, Juan Antonio Calleja, Isabel Draper, Pablo Aguado-Ramsay, Ricardo Garilleti

**Affiliations:** 1 Departamento de Botánica y Geología, Universidad de Valencia, Avda. Vicente Andrés Estellés s/n, E-46100 Burjassot, Spain; nikolay.matanov@uv.es; 2 Departamento de Biología (Botánica), Facultad de Ciencias, Universidad Autónoma de Madrid, C/Darwin 2, E-28049 Madrid, Spain; francisco.lara@uam.es (F.L.); juan.calleja@uam.es (J.A.C.); isabel.draper@uam.es (I.D.); pablo.aguador@uam.es (P.A.-R.); 3 Centro de Investigación en Biodiversidad y Cambio Global, Universidad Autónoma de Madrid, C/Darwin 2, E-28049 Madrid, Spain

**Keywords:** *Atlantichella*, Australasia, biogeography, *Lewinskya*, New Zealand, *Rehubryum bellii*, *Rehubryum kiwi*, systematics, taxonomy

## Abstract

In recent years, phylogenomic approaches have significantly deepened our understanding of moss diversity. These techniques have uncovered numerous previously overlooked species and provided greater clarity in resolving complex taxonomic relationships. In this context, the genus *Rehubryum* is particularly outstanding, because of its close morphological similarity to both *Ulota* and *Atlantichella*. The challenges posed by its segregation are addressed in this study, which integrates morphological and molecular data to reassess the circumscription of *Rehubryum* and its phylogenetic placement within the subtribe Lewinskyinae. Our results support the recognition of a new species, *R. kiwi*, and show that its inclusion within the genus further complicates the morphological delimitation of *Rehubryum* from *Ulota*, as both genera are distinguishable by only two consistent gametophytic characteristics: a submarginal leaf band of elongated cells, and the presence of geminate denticulations in the margins of the basal half of the leaf. Moreover, *R. kiwi* challenges the current morphological circumscription of *Rehubryum* itself, as it overlaps in key characteristics with its sister genus *Atlantichella*, rendering their morphological separation untenable. The striking interhemispheric disjunction between *Rehubryum* and *Atlantichella* raises new questions about long-distance dispersal and historical biogeography in mosses, despite these complexities at the generic level. Nevertheless, species-level distinctions remain well defined, especially in sporophytic traits and geographic distribution. These findings highlight the pervasive cryptic diversity within Orthotrichaceae, underscoring the need for integrative taxonomic frameworks that synthesize morphology, molecular phylogenetics, and biogeography to resolve evolutionary histories.

## 1. Introduction

Historically, the classification of bryophytes relied on comparative morphology, as reflected in traditional taxonomic frameworks, e.g., [1,2], which assumed that morphological synapomorphies accurately represented natural evolutionary lineages [3]. However, advances in molecular phylogenetic methodologies, particularly next-generation sequencing (NGS) approaches, have led to a major reorganization of bryophyte systematics [4,5]. At a finer taxonomic scale, integrative taxonomy has significantly improved the understanding of infrafamilial relationships, enabling the redefinition of previously poorly understood species groups. These advances challenge the perception of bryophyte simplicity and prompt a re-evaluation of traits formerly attributed to homoplasy [6].

A notable example of this taxonomic refinement is exemplified by the genus *Ulota* D. Mohr, from which three monotypic genera—*Plenogemma* Plášek, Sawicki & Ochyra; *Atlantichella* F.Lara, Garilleti & Draper; and *Rehubryum* F.Lara, Garilleti & Draper—have been recently segregated [7,8,9]. Among these, the only known species of *Rehubryum*—*R. bellii* (Malta) F.Lara, Garilleti & Draper—is endemic to New Zealand. Formerly classified as *Ulota bellii* Malta, this species can be readily distinguished from *Ulota* by a suite of morphological traits [10], including a submarginal band of elongated cells in the upper part of the base and lower leaf lamina. During field surveys conducted in New Zealand, we collected specimens of a new morphotype exhibiting this diagnostic character but displaying marked sporophytic differences from the sole previously known *Rehubryum* species. In addition, both the gametophytic and sporophytic characteristics of the new specimens are equally consistent with *Atlantichella*, a genus from the Northern Hemisphere [9,10]. Because assigning these specimens to either genus based solely on morphological data proved inconclusive, we conducted an integrative study, including a phylogenetic reconstruction to clarify the relationships of this newly identified potential species, and to determine its generic placement.

The integrative approach involved a comprehensive morphological study, an analysis of its distribution in relation to some ecological factors, and the application of NGS techniques using a targeted enrichment strategy with the GoFlag 408 probe set [11]. This probe set is designed to capture 408 genomic regions from 229 single-copy or low-copy nuclear genes. It has proven effective in generating genome-scale datasets that have successfully resolved phylogenetic relationships in both vascular plants [11,12,13] and non-vascular plants [4,14,15], including members of the subfamily Orthotrichoideae [9,16]. In this latter group, this approach has led to a reconfiguration of taxonomic relationships, demonstrating clear advantages over traditional methods.

A clear illustration of how methodological choice impacts phylogenetic resolution is provided by the studies of Draper et al. [8,9]. In the first study, the authors performed a phylogenetic reconstruction of the subfamily Orthotrichoideae using classical Sanger sequencing of four molecular markers. While this approach resolved many relationships at higher taxonomic levels, it failed to provide sufficient resolution within and among several genera. In contrast, Draper et al. [9] employed the GoFlag 408 probe set, resulting in a marked improvement in phylogenetic resolution at both the genus and species levels. This phylogenomic framework successfully resolved relationships that had remained unclear in the earlier analysis, particularly within the clade comprising *Pulvigera* Plášek, Sawicki & Ochyra; *Plenogemma*; and *Atlantichella*. Similarly, Aguado-Ramsay et al. [16] applied the GoFlag 408 probe set to identify enigmatic samples from the same subfamily that could not be assigned to any taxon through morphological analysis or Sanger sequencing, even when using up to seven molecular markers. Based on these demonstrated advantages in resolving intricate relationships, we adopted this phylogenomic approach to gain deeper insights into the evolutionary history and diversification patterns within this enigmatic group of bryophytes.

## 2. Results

### 2.1. Morphological Approach

Analyses of morphological variability confirmed that the new specimens exhibit a consistent set of differential characteristics, supporting the recognition of a previously undescribed species, hereby named *Rehubryum kiwi* F.Lara, Garilleti & Matanov. In addition to the aforementioned submarginal band of elongated cells, this new morphotype displays a set of significant traits that discriminate it from other similar species. The most relevant features are detailed in Table 1 and are further commented upon in the Discussion section.

The morphological examination also corroborates that *Rehubryum kiwi* has been documented from 4 localities, all on New Zealand’s South Island, whereas *R. bellii* has been reported from 21 localities nationwide. The two species co-occur at each of the *R. kiwi* sites, and this species displays basic ecological preferences entirely subsumed within those of *R. bellii*. *Rehubryum kiwi* occupies elevations of 200–400 m a.s.l., a subset of the 200–1250 m range recorded for *R. bellii*. In addition, both mosses have been collected from the bark of trunks and branches of the same phorophytes: *Pittosporum eugenioides* A.Cunn., *Hoheria lyellii* Hook f., *Coprosma* sp., *Coriaria arborea* Linds., and *Carmichaelia* arborea Druce.

### 2.2. Assembly Summary Statistics

**Figure 1:**
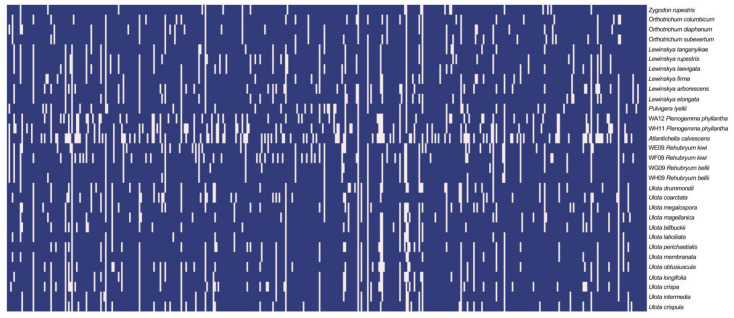
Heatmap showing the selected species versus the 398 recovered target regions used in this study. Blue indicates the successful recovery of the genetic region for the given species, while light-colored cells represent the absence of that region. Due to the large number of regions, labels on the X-axis have been omitted for clarity but are organized based on the numbering assigned during the execution of the Breinholt et al. [11] script.

Out of the 408 nuclear loci targeted by the GoFlag 408 Hyb-Seq probe set, sequences were successfully recovered for 405 target regions across the sampled species, resulting in a total of 10,903 sequences. Summary statistics are available in Supplementary Table S1. After quality filtering, 345 sequences were excluded for exceeding a threshold of >2% ambiguous sites (Supplementary Table S2). Additionally, seven regions (L73, L149, L150, L193, L213, L253, and L255) were excluded due to insufficient representation, having sequences from fewer than four species. Of the remaining 398 target regions, only 114 were represented across all 31 sampled taxa, while eight loci were recovered in fewer than ten species. Among the studied taxa, *Atlantichella calvescens* exhibited the lowest number of loci recovered, with 273 regions successfully sequenced, whereas *Zygodon rupestris* and *Ulota laticiliata* showed the highest numbers, with 376 and 374 loci, respectively. Regarding the studied individuals of *Rehubryum*, a substantial number of loci were recovered, with 345 and 335 genomic regions successfully recovered for *R. kiwi*, and 368 and 370 for *R. bellii*. A summary visualization of locus recovery across samples is shown in the heatmap (Figure 1).

The final concatenated matrix consisted of 471,531 bp, with an average missing data proportion of 39.66%. Notably, 70 out of the 398 loci had more than 39% missing data. This missing data were attributable to the inclusion of flanking regions, which varied in sequence completeness and length across species, often resulting in partial sequences at both ends of a given locus.

Despite these gaps, the dataset demonstrated a high level of parsimony-informative sites, with an average of 18.6% per locus. Only 38 loci had a proportion of parsimony-informative sites less than or equal to 10%.

### 2.3. Phylogenomic Reconstructions and Support

**Figure 2:**
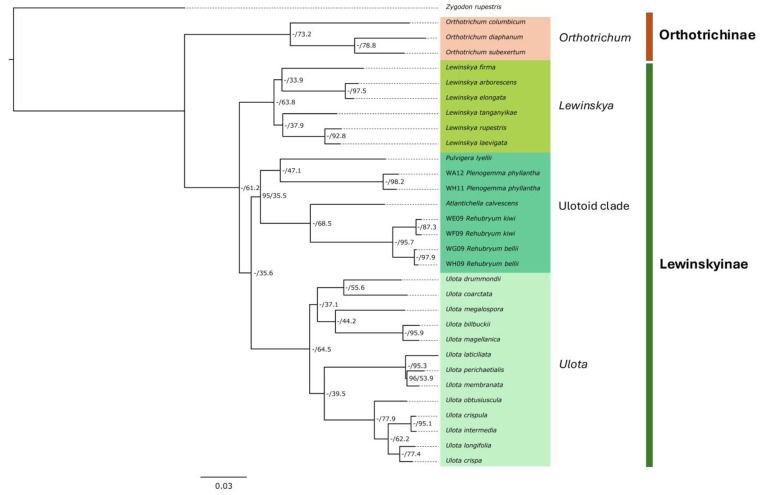
Phylogeny based on a concatenated matrix composed of 398 recovered target regions, reconstructed using IQ-TREE (partitioned with the Bayesian information criterion and incorporating the FreeRate heterogeneity model [MFP + MERGE option]). Ultrafast Bootstrap (UFBoot) values < 100% are shown at each node, whereas nodes with full support (UFBoot = 100) are indicated with a dash (−). Site concordance factor (sCF) values are provided alongside the corresponding BS values to further assess node support.

The phylogenies inferred using IQ-TREE and ASTRAL-III displayed largely congruent topologies, with minor discrepancies in node support values. The outgroup was consistently resolved with high support, and the clade of the subtribe Orthotrichinae (including *Orthotrichum columbicum*, *O. diaphanum*, and *O. subexsertum*) was recovered as a distinct sister lineage from the subtribe Lewinskyinae F.Lara, Garilleti & Draper (Figure 2). Within Lewinskyinae, three primary clades were identified: one comprises all of the species belonging to *Lewinskya* F.Lara, Garilleti & Goffinet; and one includes those of *Ulota*. The species representing *Rehubryum*, *Atlantichella*, *Plenogemma*, and *Pulvigera* are gathered in a clade already identified by Draper et al. [9], henceforth designated as the ulotoid clade, due to the morphological resemblance of most of its genera to *Ulota*. *Rehubryum* specimens were consistently recovered as monophyletic and formed a sister group to *Atlantichella*. Together, these two genera were resolved as sisters to a clade comprising *Plenogemma* and *Pulvigera*, recovering the same topology as in the work of Draper et al. [9].

**Figure 3:**
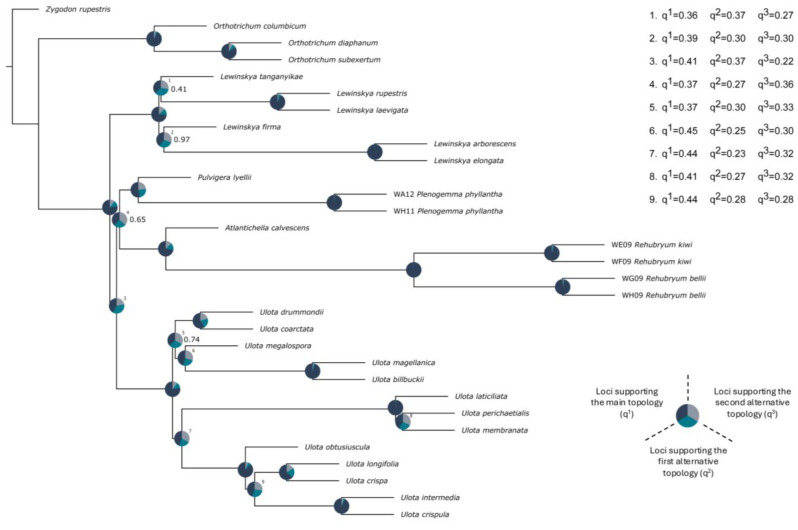
Phylogeny based on the coalescence of 398 gene trees from the recovered target regions, reconstructed using ASTRAL-III. Branches with LPP values < 1 are noted. Pie charts at nodes illustrate gene tree conflict evaluations, showing the proportion of gene trees in concordance with the main topology (q^1^), the first alternative topology (q^2^), or the second alternative topology (q^3^). Nodes where the main topology is represented by <50% of gene trees are numbered, and their q^1^, q^2^, and q^3^ values are indicated.

Most nodes in the ML phylogeny exhibited robust support, with fully supported UFBoot values. However, the basal node within the ulotoid clade showed slightly lower support (UFBoot = 95), as did the relationship between the two endemic New Zealand species *U. membranata* and *U. perichaetialis* (Figure 2). In contrast, the coalescent analyses yielded lower LPP across a greater number of nodes (Figure 3). Despite these differences, both approaches supported the separation of the ulotoid clade from *Lewinskya* and *Ulota* and consistently resolved the newly identified morphotype as a distinct, well-supported lineage within the genus *Rehubryum*, clearly separated from its sister species *R. bellii*. Notably, nodes exhibiting lower site concordance factor (sCF) values corresponded to those with the highest topological conflict in the coalescent-based analyses, suggesting localized phylogenetic discordance at those points in the tree. Nevertheless, none of the sCF values across the tree fell below 33%, the theoretical expectation under random site support among three possible topologies [17]. This indicates that, despite some localized conflict, there is no evidence of pervasive site-level noise or lack of phylogenetic signal. On the contrary, the consistently moderate-to-high sCF values across all nodes suggest a generally strong underlying phylogenetic structure, reinforcing the reliability of the main topological conclusions obtained through both ML and coalescent approaches.

### 2.4. Taxonomy

#### *Rehubryum kiwi* F.Lara, Garilleti & Matanov sp. nov. (Figure 4 and Figure 5)

Type: New Zealand, South Island, West Coast Region, Westland District, Fox Glacier, shrubland above the carpark, 43°29′47′′ S, 170°02′37′′ E, 240 m a.s.l.; shrubland dominated by *Coriaria*, *Olearia*, *Carmichaelia*, and *Aristotelia*, on *Coriaria arborea*, 29 January 2016, *R. Garilleti 2016-102c & F. Lara* (Holotype BM, Isotypes MAUAM, Herb. Garilleti).

Plants small, up to 1.8 cm, forming cushions up to 3 cm wide, pale green to pale brown, of strongly crisped appearance when dry. Stems pentagonal to circular in section, with scleroderm scarcely differentiated, formed by 2–3 layers of small cells with thickened walls, and parenchyma with bigger cells and moderately thickened walls, without central strand. Axillary hairs up to four cells high, hyaline, with the basal cell colored and much shorter. Rhizoids reddish to orange, smooth, and restricted to stem base. Flagelliform branches or stolons not seen. Leaves circinate when dry, linear–lanceolate, erect–patent when moist, (1.7–)2.0–3.0 × 0.4–0.9 mm, with base oval; leaf apex acuminate to acute, frequently ending in one apical cell; leaf base ellipsoidal to obovate, ± concave, gradually narrowing into a linear to linear–lanceolate and variably keeled lamina; costa almost reaching the leaf apex, 30–54 μm wide at leaf base, (21–)27–40(–50) μm wide at mid-leaf; margins plane, below denticulated by geminate papillae arising at the junction between marginal cells, especially at the base–lamina transition, entire the rest of the lamina, occasionally slightly crenulate near the apex; lamina unistratose; upper and median lamina cells isodiametric, oval, or oblate, (6–)7–15(–18) × (4–)5–11 μm, smooth or with one or two very low papillae; basal cells elongate to closely rectangular–ellipsoidal, with thick sinuous walls, 20–57 × 4–10 μm, wider and orange–red colored near leaf insertion; submarginal cells differentiated in a band of (2–)3–4(–6) rows of elongated cells, from the upper part of the base through the lower 1/4 to 1/3 of the lamina; basal marginal cells differentiated in (2–)4–8 rows, rectangular and hyaline with thickened transverse walls. Brood bodies absent.

Goniautoicous, perigonia lateral, gemmiform, perigonial leaves ovate, acute, 0.6–0.9 × 0.4–0.6 mm. Perichaetia terminal, perichaetial leaves differentiated, longer and wider than the vegetative ones, 2.5–3.5 × 0.5–0.9 mm, lanceolate, gradually tapering towards an acute to acuminated apex, with subpercurrent costa. Vaginula cylindrical to oval–cylindrical, 0.45–0.75 mm long, sparsely hairy, hairs thin, fragile, yellowish to coppery, uniseriate to partially pluriseriate, papillose. Calyptra mitrate, 1.5–2.0 mm long, yellowish to light orange with brown beak, ± densely hairy with thick, pluriseriate, papillose hairs reaching the beak. Sporophytes commonly present. Seta (3.0–)3.5–5.0(–6.0) mm long, reddish, twisted counterclockwise when dry. Capsules long-exerted, (1.0–)1.3–2.0(–2.3) mm long, pale brown, ellipsoidal to globose when wet, ovoid when dry and full of spores, short cylindrical when dry and empty, sulcate along the entire length by eight prominent ribs; neck 0.3–0.6 mm long; mouth rounded, often irregular; exothecial cells quadrate to rectangular, occasionally sinuous; eight exothecial bands, differentiated from mouth to urn base, of 2(–3) rows of cells with thickened longitudinal walls, concolor with exothecial cells at base, turning darker upwards and becoming crimson near the mouth; suboral cells arranged in three or four rows of isodiametric cells with crimson, thickened walls. Stomata phaneroporous, restricted to urn base and neck. Operculum convex to conical and rostrate, with broad, crimson basal rim, 0.4–0.5 mm in diameter. Peristome double. Prostome absent. Exostome of eight pairs of teeth, sometimes partially split in mature capsules, fenestrated at tips, revolute when dry, pale orange below and cream-colored towards apex, (180–)220–320(–350) μm long; outer peristome layer (OPL) papillose, with papillae more prominent towards the tip, exostomial primary peristome layer (PPL) with longitudinal lines at basal half, distally ornamented by dense papillae, sometimes forming lines, with marked trabeculae at tips. Endostome of eight linear segments, not widened at base, fragile, two-thirds as long as exostome teeth, hyaline and shiny, uniseriate, sometimes with irregular remains of a second row of cells, incurved to involute when dry, 130–200 μm long; endostomial PPL smooth; inner peristome layer (IPL) papillose, with thin trabeculae; connecting membrane present, hyaline, located at the base of the teeth and hardly noticeable. Spores spherical to irregularly ovoid, with thick papillae sometimes forming verrucae, orange to light brown, (17–)20–28(–34) μm.

## 3. Discussion

### 3.1. Systematic and Taxonomical Delimitation

Recent advances in genomic research have significantly reshaped our understanding of bryophytes’ diversity and phylogenetic relationships. High-resolution genomic data have uncovered substantial hidden diversity by revealing cryptic lineages, suggesting that global bryophyte biodiversity is considerably underestimated [4]. These findings challenge traditional morphological classifications, as many traits previously considered to be diagnostic at the generic or familial level are now recognized as homoplastic, resulting from convergent evolution or evolutionary reversals [18]. Consequently, there is a growing consensus on the necessity of integrating genomic evidence with detailed morphological analyses to refine taxonomic boundaries and better reflect true phylogenetic relationships within bryophytes.

Within Orthotrichoideae, recent phylogenomic studies have resolved three ulotoid genera—*Plenogemma*, *Atlantichella*, and *Rehubryum*—as distinct lineages derived from a common ancestor [8,9]. Traditionally, these three genera were included within the genus *Ulota*, as all four genera share a suite of morphological traits once thought to be diagnostic at the generic level [19]. For instance, they have a crisped appearance when dry, and their leaves possess a band of hyaline basal marginal cells with thickened transverse walls, clearly differentiated from the other basal leaf cells. However, they differ from *Ulota* by having a submarginal leaf band of elongated cells, rising some distance from the base–lamina junction, together with geminate denticulations along the margins of the basal half of the leaf. Among these ulotoid genera, *Plenogemma* is readily identifiable by its dioicous condition and the presence of apical gemmae tufts, whereas *Atlantichella* and *Rehubryum* are more difficult to distinguish from *Ulota*, differing only in the aforementioned leaf areolation features.

Until now, *Rehubryum* was considered to be a monotypic genus morphologically circumscribed exclusively by the traits of *R. bellii.* The discovery of *R. kiwi* broadens the morphological scope of the genus and renders the separation from its sibling genus, *Atlantichella,* even more ambiguous. The number of endostomial segments can no longer be considered differential [10], as *R. kiwi* and *A. calvescens* share the possession of only eight segments. Consequently, the size and shape of the dried capsules are the only observable morphological distinctions between *Rehubryum* and *Atlantichella* (Figure 6). Therefore, any attempt to morphologically circumscribe *Atlantichella* and *Rehubryum* becomes untenable, as no exclusive characteristics remain to reliably delimit them within the subtribe Lewinskyinae. This lack of exclusive characteristics to separate genera has been previously noted and discussed within the family Orthotrichaceae [8], as well as in other families of mosses, such as Brachytheciaceae [20] or Funariaceae [21], and is interpreted as a generalized homoplasy in key morphological characteristics.

The widespread occurrence of homoplastic characteristics in Orthotrichaceae highlights the necessity of adopting a comprehensive and integrative approach to understanding species boundaries and phylogenetic relationships. Moreover, the absence of autapomorphic traits significantly complicates the accurate delimitation of genera, reinforcing the importance of molecular data in resolving taxonomic uncertainties. The phylogenetic reconstructions achieved (Figure 2 and Figure 3) strongly support the species-level status of *Rehubryum kiwi*, with full support of its divergence from its sister species *R. bellii* and their separation from *Atlantichella calvescens*.

Nevertheless, despite the lack of genus-level morphological characteristics, the three species currently assigned to *Atlantichella* and *Rehubryum* can be readily distinguished from each other based on species-specific morphological features and their widely disjunct geographic distributions. Morphological differentiation among these species is primarily restricted to the sporophyte (Table 1, Figure 6), while the gametophyte exhibits only minor, non-diagnostic variation.

The western Palearctic species, *Atlantichella calvescens*, is distinctive for its long urceolate capsules, broadly ribbed and strongly contracted below the mouth when dry and empty. Additionally, its exostome quickly splits into 16 teeth. The large size of the sporophytes (up to 8 mm in length) is also a characteristic of the species [22]. However, the most distinctive features lie in the structure of the exothecial bands, composed of (3–)4–5 rows of cells, and the prominent longitudinal ribs, which leave little or no space between them when dry. These ribs may appear variably darkened but remain concolorous with the adjacent intercostal area.

*Rehubryum bellii* is clearly distinguished from the other two species by its conspicuous endostome, consisting of 16 segments of equal length, arising from a high connective membrane that extends beyond the capsule mouth. By contrast, both *R. kiwi* and *A. calvescens* possess only eight segments, with a low connective membrane, which in *A. calvescens* is often discontinuous.

*Rehubryum kiwi* is also recognizable by its short cylindrical capsules with thin but distinctly prominent, reddish-pigmented ribs, which are a consequence of the strongly differentiated exothecial bands composed of 2(–3) rows of cells. Moreover, its exostome is characterized by paired teeth that typically remain united, with only occasional instances of partial separation observed.

Traits such as capsule shape, number of exothecial bands, and endostome architecture have already been demonstrated as useful for discriminating closely related species in other genera within Orthotrichaceae, such as *Ulota* [23,24,25,26,27] or *Pulvigera* [28].

Finally, it should be noted that *Rehubryum kiwi* exhibits a narrower chorological and ecological range than *R. bellii*. *Rehubryum kiwi* is confined to four South Island localities at 200–400 m a.s.l., whereas *R. bellii* occupies 21 sites across both main islands and spans 0–1250 m. However, the two mosses always co-occur at the *R. kiwi* sites and share the same phorophyte hosts. Hence, *R. kiwi* appears to occupy a niche nested within that of *R. bellii*. Phylogenetic closeness consistently entails a pattern of marked similarity in both chorology and ecology [29] that has been previously documented in other mosses, including epiphytic members of the Orthotrichaceae [30]. Sister taxa that are morphologically very similar can coexist within the same locality, and even on the same phorophyte, without showing signs of hybridization [31,32].

The case of *Rehubryum kiwi* and *R. bellii* raises unresolved questions about the mechanisms and timing of their speciation and the nature of the reproductive barriers that keep them distinct. *Rehubryum kiwi* does not appear to have undergone ecological speciation [33], as it shows no adaptation to different macro- or microenvironmental conditions. As inferred for other plants, *R. kiwi* may have diverged from *R. bellii* during a period of historical geographic isolation (i.e., geographical speciation), during which unidentified reproductive barriers arose, and later come into secondary contact [33]. Alternatively, in bryophytes, reproductive barriers may occur in more parsimonious scenarios, from intrinsic mechanisms alone or from combinations of intrinsic and extrinsic factors [34]. For instance, seasonal differences in the maturation of male and female gametangia between species can prevent interspecific fertilization [35]. However, the specific barriers acting in *Rehubryum* have yet to be identified.

### 3.2. Phylogeographical Interpretation of Rehubryum Origin

**Figure 4:**
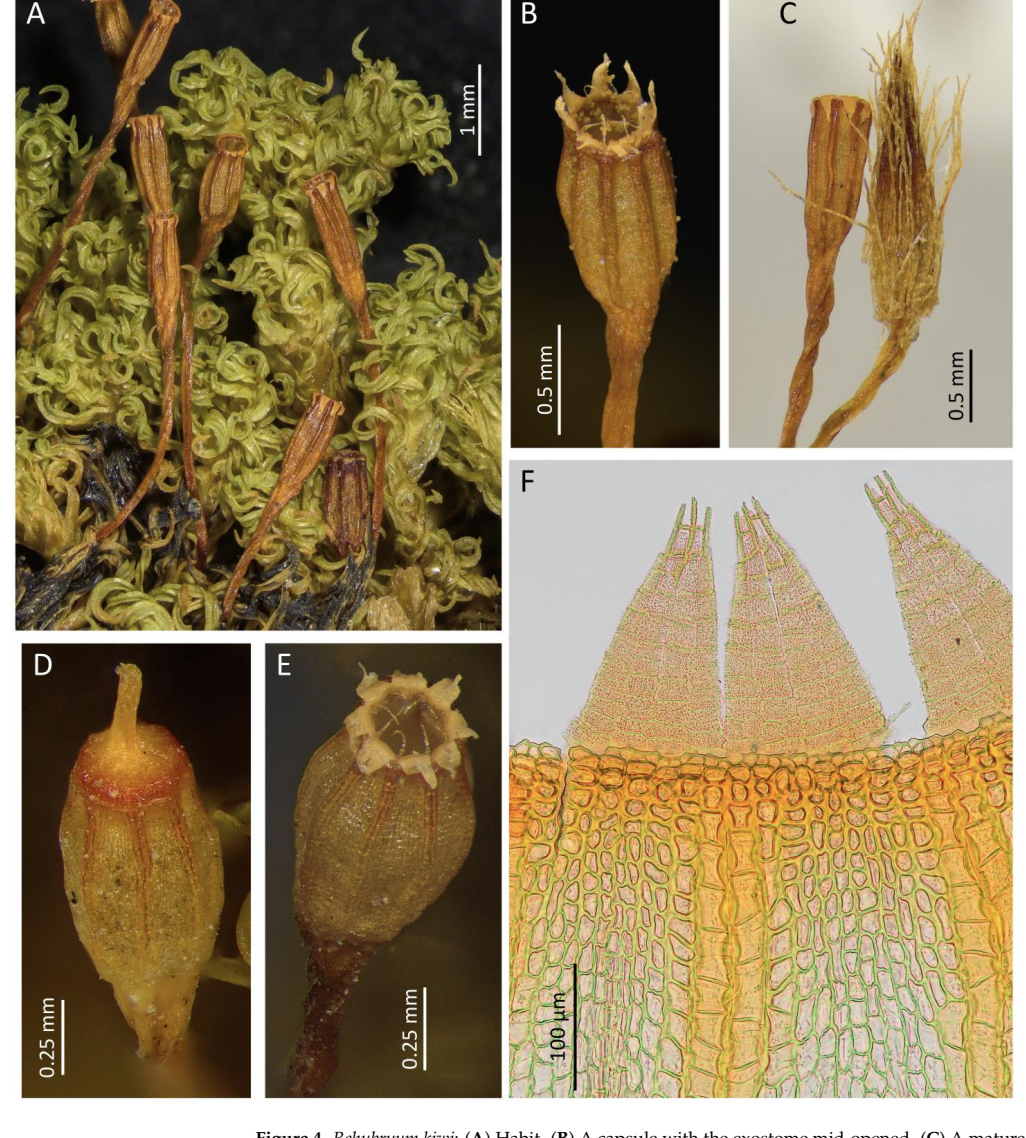
*Rehubryum kiwi*: (**A**) Habit. (**B**) A capsule with the exostome mid-opened. (**C**) A mature capsule and a young one, with calyptra. (**D**) An operculate capsule. (**E**) A mature capsule; note the thin, colored exostomial bands and the 8-segment endostome. (**F**) Detail of a capsule with 2-cell-wide exothecial bands and exostome with teeth in pairs. ((**A**) Lara 1601/148; (**B**) Garilleti 2016-104b; (**C**) Garilleti 2016-102c; (**D**–**F**) Lara 2001 s.n.)).

**Figure 6:**
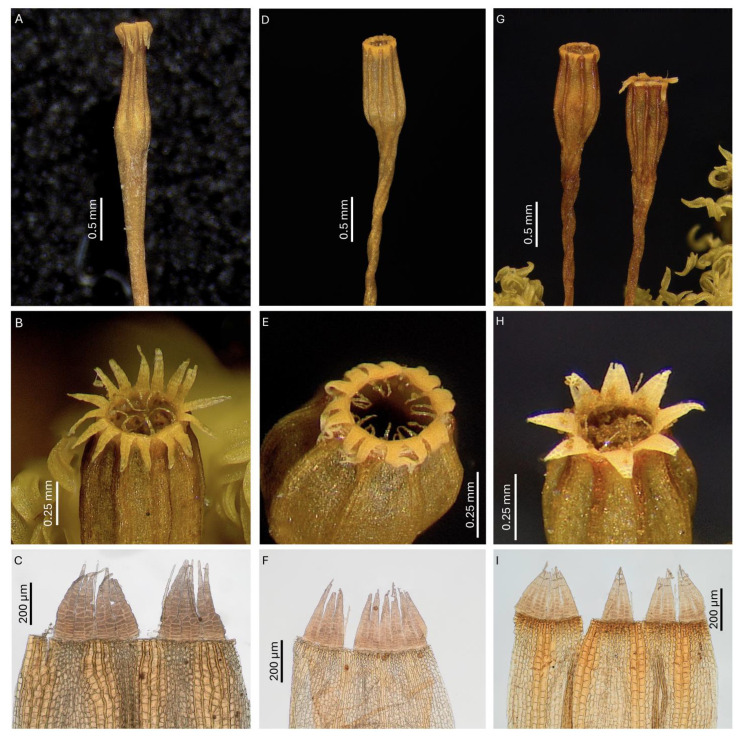
Comparison between *Atlantichella* and *Rehubryum*: (**A**–**C**) *A. calvescens*; (**D**–**F**) *R. bellii*; (**G**–**I**) *R. kiwi*. (**A**,**D**,**G**): Capsule shape; note the differential constriction in *Atlantichella*. (**B**,**E**,**H**): Peristomial constitution; *R. bellii* is the only species with a 16-segment endostome. (**C**,**F**,**I**): Detail of exothecium and peristome; note the different configuration of the exothecial bands, wider and most developed in *Atlantichella*, and thinner and brightly colored in *R. kiwi*. ((**A**) MUAM-Brio 4442; (**B**) Garilleti 2016-138; (**C**) Garilleti 2016-142; (**D**) Garilleti 2016-041a; (**E**) Garilleti 2016-030; (**F**) Lara 1601-64; (**G**) Garilleti 2016-104b; (**H**) Lara 2001 s.n.; (**I**) Garilleti 2016-102c).

The obtained phylogenomic results place the ulotoid clade within the tribe Lewinskyinae, consistently recovering a sister relationship between *Rehubryum* and *Atlantichella*, which together form a clade with *Pulvigera* and *Plenogemma*. However, the precise relationships among these genera remain somewhat unresolved, as evidenced by the low node support in both concatenation and coalescent analyses. This is likely attributable to gene tree conflict, particularly at nodes where alternative topologies receive nearly equal support (Figure 4). This conflict may arise from several sources, including hidden paralogy [36,37], hybridization [38], or incomplete lineage sorting (ILS) [39,40]. The equal distribution of quartet scores suggests that ILS is the most probable cause here. In contrast, hidden paralogy is unlikely in this case, because the sequences obtained for each of the targeted loci were clear within each of the studied species and could be unambiguously mapped against reference genomes from mosses, hornworts, liverworts, lycophytes, ferns, and gymnosperms to confirm orthology. Finally, reticulation events typically generate two strongly supported topologies against a third with lower support [41], a pattern not clearly observed here. Nevertheless, given that some values indicate a slight tendency toward supporting two topologies over a third, we cannot entirely rule out the possibility of ancient reticulation events, particularly at the basal node of the ulotoid clade (which shows supports of q^1^ = 0.37, q^2^ = 0.27, and q^3^ = 0.36, and also displayed site concordance factor (sCF) values below 50%). The sCF values in this range suggest that a substantial proportion of informative sites may support conflicting topologies, potentially reflecting underlying genealogical discordance [17]. While this alone does not confirm reticulation, it is consistent with patterns expected under incomplete lineage sorting and, to a lesser extent, ancient hybridization, especially when combined with balanced quartet scores and reduced sCF values. Also, as Hibbins & Hahn [42] have shown, signals of ancient reticulation can be eroded over time by recombination, selection, or lineage extinction, which may help in explaining the diffuse patterns observed in our dataset.

We interpret that the suggested ILS may have resulted from rapid radiations, in which polymorphic loci present at the time of lineages’ divergence would have become randomly fixed [43]. This stochastic fixation could have led to a pattern that does not accurately reflect the true evolutionary history, resulting in discordance between the species tree and the individual gene trees [44].

The vicariance involving *Atlantichella* and *Rehubryum* is particularly noteworthy, as these sibling genera exhibit a perfectly antipodal distribution. Although the exact timing of their divergence from a common ancestor remains undetermined, it may have been relatively recent, between 35 and 25 Ma, based on the diversification of *Ulota* and the ulotoid *Pulvigera* estimated by Draper et al. [8], or the divergence of *Plenogemma* and *Pulvigera* estimated by Bechteler et al. [4]. However, this interpretation should be taken with caution, as neither of these studies included *Rehubryum*, and the topology of the dated trees slightly differed from that obtained using Hyb-Seq by Draper et al. [45], where the phylogenetic relationships of the ulotoid clade were defined. Consequently, although the antipodal distribution of these genera suggests a major vicariant event, the absence of direct temporal and phylogenetic evidence leaves an enigma about the biogeographic processes involved.

Disjunctions in bryophytes are more frequently observed within the same hemisphere. It has been hypothesized that the distribution of landmasses and wind currents favors long-distance dispersal in both hemispheres [31,46]. Bipolar disjunctions are less common and typically involve species that are widely distributed in the Northern Hemisphere, where they also exhibit disjunct areas from which they have expanded southward [47,48,49,50]. Latitudinal movements are more frequent within relatively continuous landmasses [47,48,50], and long-distance dispersal followed by diversification is often interpreted as occurring from the Northern to the Southern Hemisphere [51,52]. However, in some cases, bidirectional dispersal and taxa segregation within the same genus have been documented [49]. Ochyra & Buck [52] summarize three possible trans-tropical dispersal tracks that could facilitate such movements: (i) the Cordilleran route, spanning the American continent down to Patagonia and the maritime Antarctic; (ii) the Indomalayan–Melanesian pathway, extending across the Malesian island chain to southeastern Australia, New Zealand, and nearby archipelagos; and (iii) the African route, crossing the East and South African mountains to the sub-Antarctic islands.

The African dispersal route is approximately in line with the Rand Flora pattern described for seed plants, which accounts for the distribution of closely related lineages along the peripheral mountain ranges of the African continent, including the Mediterranean Basin and Macaronesia, and reaching South Africa [53]. This biogeographic pattern would theoretically allow for the dispersal between hemispheres and subsequent movement to Australasia of a hypothetical ancestor of *Rehubryum* and *Atlantichella*. An alternative scenario is based on the strong bryofloristic connections between Patagonia, the sub-Antarctic islands, and Australasia, which have long been recognized, presumably facilitated by prevailing sub-Antarctic wind systems [46,54,55,56,57]. This hypothesis is supported by the greater role of long-distance dispersal in the Southern Hemisphere in seed plants, compared to processes derived from drift patterns [58,59]. Such enhanced mobility via southern winds would allow the mobility of plants previously using the Cordilleran route of Ochyra and Buck [52] for interhemispheric migration. However, there is currently no evidence supporting the presence of a direct ancestor of *Atlantichella* or *Rehubryum* on the American continent.

Mountainous regions in Africa may have provided suitable conditions for *Ulota* and ulotoid species since the Paleocene [60], as observed in present-day tropical regions worldwide, including cool periods in the Early Oligocene. This period was also accompanied by the early diversification of vascular flora in West Africa [61], which is particularly relevant given the epiphytic nature of these ulotoid species. Once reaching the southernmost extent of the continent, dispersal to Australasia via the prevailing westerly winds appears plausible. Nevertheless, this Africa–Australasia pattern has not traditionally been incorporated into biogeographical models addressing connectivity among austral landmasses [46,55].

Nonetheless, the precise dispersal route remains unresolved. As a moderately sized archipelago and a remnant of the submerged continent Zealandia, New Zealand exhibits diverse biogeographical patterns [62]. Climatic oscillations and geological events, including the rapid uplift of mountain ranges during the Pliocene and Pleistocene, have profoundly influenced its contemporary biota [63]. Based on all of this evidence, and considering the epiphytic and ecological traits of the species, our findings align with the hypothesis of a recent rapid dispersal of the ancestor of *Rehubryum*. Thus, the detected phylogenetic connection can be ruled out as a consequence of an originally continuous area in ancient scenarios (e.g., Gondwana) that was later fragmented by continental drift [64].

In summary, the common ancestor likely had a widespread distribution, but subsequent extinction, speciation, and long-distance dispersal events must have led to contractions and expansions of its range. Additional evidence for similar biogeographic patterns can be found in other bryophyte taxa occurring in both South Africa and Australia, lending further support to this hypothesis [47,65]. Notable cases include *Archidium rehmannii* Mitt., *Ephemerum capense* Müll.Hal., *Bartramia breutelii* Schimp. ex Müll.Hal., or the genus *Stoneobryum* D.H.Norris & H.Rob., which comprises only two species: *Stoneobryum bunyaense* D.H.Norris & H.Rob. from Australia, and *Stoneobryum mirum* (Lewinsky) D.H.Norris & H.Rob. from South Africa [66]. Even *Orthodontium lineare* Schwägr. exhibits a remarkably disjunct distribution, occurring in Central Europe, Southern Africa, Australia, and Patagonia.

## 4. Materials and Methods

### 4.1. Taxon Sampling

**Figure 7:**
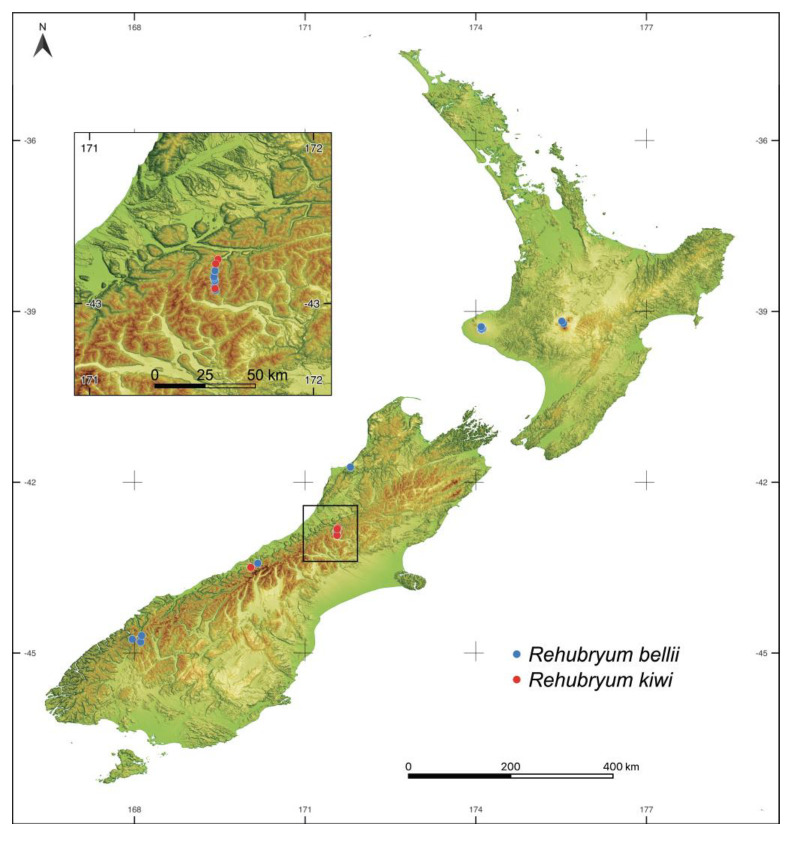
Sampled localities across the North and South Islands of New Zealand where *Rehubryum bellii* and the new species *R. kiwi* were observed. Map created with QGIS 3.22.6 (available at www.qgis.org, accessed on 15 April 2022); boundaries, rivers, and DEM data downloaded from the Land Information New Zealand (LINZ) Data Service (https://data.linz.govt.nz, accessed on 14 November 2022), with a Creative Commons Attribution 4.0 International license.

This study is based on specimens collected during two field expeditions to New Zealand in the austral summers of 2000–2001 and 2016. Sampling encompassed 96 localities across the North Island (34 sites) and South Island (62 sites), spanning an altitudinal gradient from sea level to 1430 m above sea level (for a complete map of the sampled localities, see Ref. [10]). The sampling sites were strategically selected to target ecologically favorable habitats for mosses of the subfamily Orthotrichoideae. At each site, the basic attributes were recorded, including the sampled phorophytes and the microsites where the specimens were found (trunks, branches, etc.). The newly identified morphotype was observed at only four localities (Figure 7). All of the found specimens of the target morphotype, together with 19 specimens of *Rehubryum bellii* from both islands and 4 specimens of *Atlantichella calvescens* from other collections in Europe, were included in the morphological analyses. For DNA extraction, two specimens of the target morphotype and two of *R. bellii* were selected based on their quality and preservation. Additionally, newly collected voucher specimens of *Plenogemma phyllantha*, *Ulota laticiliata*, *U. billbuckii*, and *U. magellanica* were incorporated to broaden the taxonomic representation in the analyses. All of the samples included in the phylogenetic analysis are listed in Supplementary Table S3.

### 4.2. Morphological Analyses

Morphological examination was conducted using a combination of macroscopic and microscopic characteristics, selected based on our previous work on Orthotrichaceae [10,67,68]. A total of 57 gametophytic and 71 sporophytic traits were examined across all of the selected specimens, encompassing key morphological features relevant to taxonomic differentiation [69].

### 4.3. DNA Extraction

For the selected samples, we followed a modified cetyltrimethylammonium bromide (CTAB) extraction protocol [70], as outlined by Breinholt et al. [11]. Tissue homogenization was performed using a Geno/Grinder 2010 mill (SPEX CertiPrep, Metuchen, NJ, USA). Two rounds of 24:1 (*v*/*v*) chloroform–isoamyl alcohol washes were conducted, followed by cold isopropanol precipitation and a final wash with 70% (*v*/*v*) ethanol. To eliminate RNA contamination, 2 µL of 10 mg/mL RNase A (QIAGEN, Valencia, CA, USA) was added between the chloroform washes.

### 4.4. Target Enrichment and Sequencing Assembly

A dataset comprising 31 accessions was compiled by integrating previously published target enrichment data from Draper et al. [9] with newly generated data from this study (Supplementary Table S3). Thus, the final dataset included representatives from seven of the ten genera within the tribe Orthotricheae, encompassing 27 species in total, including representatives from all genera within the subtribe Lewinskyinae, to which *Rehubryum* and its related genera belong. Specifically, it comprised *Atlantichella calvescens* (100% of the genus), six species of *Lewinskya* (ca. 10% of the genus), two samples of *Plenogemma phyllantha* (100% of the genus), *Pulvigera lyellii* (25% of the genus), thirteen species of *Ulota* (ca. 20% of the genus), and, along with two samples of the new morphotype, two samples of *Rehubryum belli* (100% of the genus). Additionally, three *Orthotrichum* species were included to enhance the phylogenetic reconstruction with representatives of the subtribe Orthotrichinae, and one representative of the tribe Zygodonteae, *Zygodon rupestris*, was included to root the tree.

Target capture sequencing of DNA from newly sampled accessions was performed at RAPiD Genomics (Gainesville, FL, USA) using the GoFlag 408 probe set for flagellate land plants [11]. Raw Illumina sequence data were processed through the GoFlag pipeline, as described by Breinholt et al. [11]. Adapters and low-quality bases were trimmed from raw reads using Trim Galore! version 0.6.10 [71], and target regions were assembled via an iterative baited assembly (IBA) approach [72]. The IBA workflow utilized USEARCH version 7.0 [73] to identify homologous matches to reference sequences, followed by de novo assembly for each locus with BRIDGER version 2014-12-01 [74], using three iterations, a k-mer size of 25, and a minimum coverage threshold of 10 nucleotides.

Orthologous sequences were identified and formatted for downstream analyses using tBLASTx from BLAST + version 2.15.0 [75] against ten flagellate land plant genomes [11]. To maintain data integrity, sequences matching to reference genomes from clades outside the moss lineage were excluded to prevent contamination. Final sequence alignments for each locus were generated using MAFFT version 7.490 [76]. For samples with multiple sequences per locus in the alignment, putative isoforms were merged, with nucleotide ambiguity codes representing variable sites. Samples with multiple unresolved sequences for a given locus were subsequently removed from the dataset.

### 4.5. Species Tree Inference and Evaluation of Support

Phylogenetic analyses were conducted using targeted regions, including flanking regions. Sequences were filtered based on quality and representation per genomic locus. Loci represented by fewer than four sequences and sequences containing more than 2% ambiguities were excluded. The percentage of ambiguities was calculated using AMAS [77]. Further, alignment positions with fewer than four nucleotides were removed using the filtermissing.pl script from Breinholt et al. [11]. This process minimized the impact of large gaps and highly variable regions on the downstream analyses. To visualize data completeness, a custom Python (Python 3.11.7) script was used to generate a species-by-loci matrix, which was plotted as a heatmap using the pheatmap [78] package in R.

Two complementary approaches were employed for tree estimation: a concatenation-based method, and a summary coalescent-based approach. For the concatenation-based analysis, maximum likelihood (ML) phylogenies were reconstructed in IQ-TREE v2.2.0 [79] using the concatenated gene matrix. The ModelFinder algorithm with the parameter -m MFP + MERGE [80] was applied to determine the optimal partitioning scheme, merging partitions with similar evolutionary patterns. Clade support was assessed using ultrafast bootstrap (UFBoot) analyses [81] with the parameter -bnni to reduce the risk of overestimating branch supports, incorporating 1000 UFBoot replicates.

For the coalescent-based approach, gene trees for individual loci were constructed in IQ-TREE v2.1.13 with extended model selection, -m MFP [80], and 1000 UFBoot replicates. Low-support branches (BS < 33%) were collapsed using TreeCollapse (http://emmahodcroft.com/TreeCollapseCL.html, accessed on 24 February 2023). The resulting unrooted gene trees were used to infer a species tree with ASTRAL-III v5.6.3 [82]. Node support was quantified using local posterior probability (LPP) [83], and quartet support was analyzed to evaluate gene tree conflict. Pie charts illustrating quartet support were generated using the phytools package [84] in R.

### 4.6. Studied Specimens (Paratypes)

New Zealand: South Island, Canterbury Region, Selwyn District, Arthur’s Pass National Park, Otira Valley Track parking area, 42°48′11′′ S, 171°34′25′′ E, 350 m a.s.l., bushes and small trees on gravel bank, on *Coprosma* sp., 23 January 2016, *F. Lara 1601/95 & R. Garilleti* (MAUAM). Selwyn District, Arthur’s Pass National Park, área de acampada a 1 Km del pueblo, 42°56′02S′′, 171°33′36′′, 800 m a.s.l., on *Hoheria lyallii*, 5 January 2001, *F. Lara & E. San Miguel s.n.* (MAUAM). West Coast Region, Westland District, Arthur’s Pass National Park border, Otira Hwy (73) ca. Otira town, 42°49′21′′ S, 171°33′48′′ E, 370 m a.s.l., bushes and small trees on gravel bank, on *Pittosporum eugenioides*, 23 January 2016, *F. Lara 1601/96 & R. Garilleti* (MAUAM). Westland District, Fox Glacier, shrubland above the carpark, 43°29′47′′ S, 170°02′37′′ E, 240 m a.s.l., shrubland dominated by *Coriaria*, *Olearia*, *Carmichaelia* and *Aristotelia*, on *Carmichaelia arborea*, 29 January 2016, *R. Garilleti 2016-104b & F. Lara* (Herb. Garilleti); *ibidem*, on *Carmichaelia arborea*, 29 January 2016, *F. Lara 1601/148 & R. Garilleti* (MAUAM); *ibidem*, on *Coriaria arborea*, 29 January 2016, *F. Lara 1601/146 & R. Garilleti* (MAUAM)

## 5. Nomenclatural Coda

The subtribe Lewinskyinae was invalidly published [8], since it lacked a description or reference to a previously published description or diagnosis and, therefore, required validation. Due to the segregation of this subtribe, it is also necessary to clarify the circumscription of the subtribe Orthotrichinae.

Orthotrichinae Müll. Hal. Syn. Musc. Frond. 1: 665. 1849.

Plants of small or medium size, acrocarpous, with erect stems, without stoloniform branches, often propaguliferous; monoecious; usually with terminal or pseudolateral perigonia; capsules cryptoporous or phaneroporous.

Type: *Orthotrichum* Hedw. Species Muscorum Frondosorum 162. 1801.

Genera: *Orthotrichum, Nyholmiella* Holmen & E.Warncke, *Sehnemobryum* Lewinsky & Hedenäs, and *Stoneobryum* D.H.Norris & H.Rob.

Lewinskyinae F.Lara, Garilleti & Draper, subtribe nova [*Lewinskyinae* F.Lara, Garilleti & Draper, *Frontiers in Plant Science* 12(629035): 15. 2021, nom. nud., nom. inval. ICN Art. 38.1].

Plants medium-sized, rarely small, acrocarpous, with erect or more rarely creeping stems, often with stoloniform branches, rarely propaguliferous; autoicous or rarely dioicous, usually with lateral perigonia; capsules phaneroporous.

Type: *Lewinskya* F.Lara, Garilleti & Goffinet in Lara et al. *Cryptogamie, Bryologie* 37(4): 365. 2016.

Genera: *Atlantichella*, *Lewinskya*, *Pulvigera*, *Plenogemma*, *Rehubryum*, and *Ulota*.

## Figures and Tables

**Figure 5:**
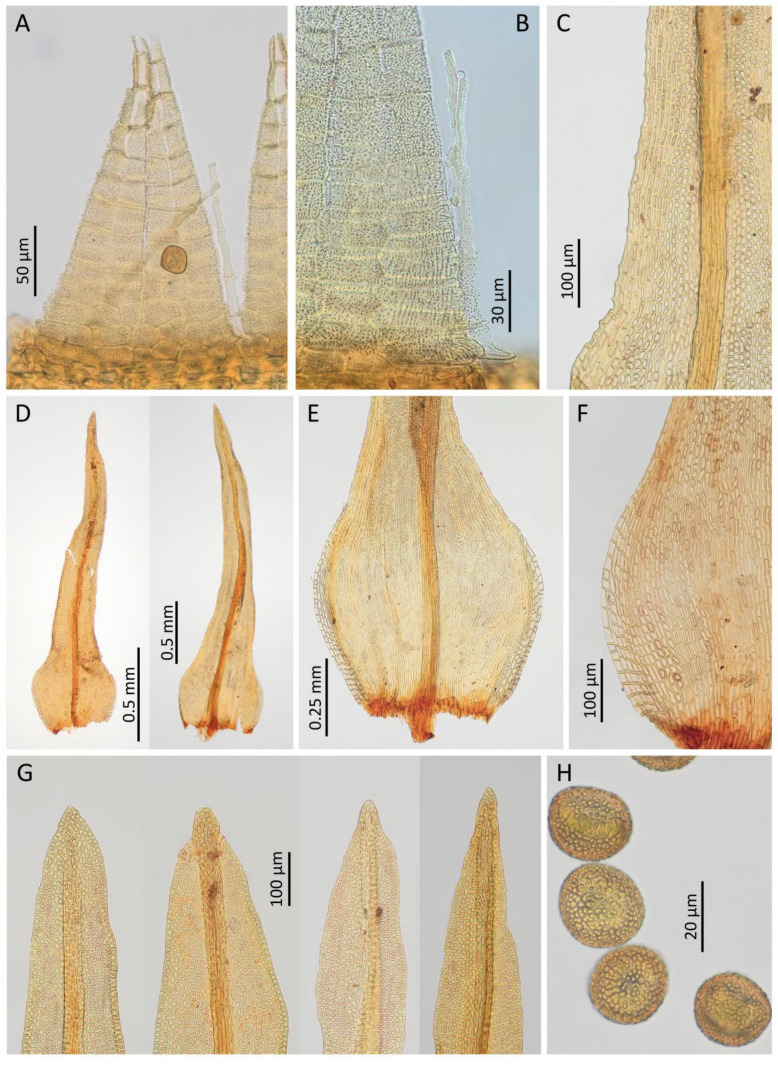
*Rehubryum kiwi*: (**A**) Detail of a pair of exostome teeth. (**B**) Endostome segment and part of a exostome tooth. (**C**) Detail of a vegetative leaf where the inframarginal band of elongated cells and the protruding marginal papillae can be observed. (**D**) Vegetative leaves. (**E**) Base of a vegetative leaf. (**F**) Detail of the marginal band of differentiated cells. (**G**) Variability in apices’ shape and costa ending. (**H**) Spores. ((**A**) Lara 2001 s.n.; (**B**–**G**) Garilleti 2016-102c; (**H**) Lara 1601/148)).

**Table 1:** Set of diagnostic morphological characteristics distinguishing *Rehubryum kiwi* from its closest morphological relatives, *R. bellii* and *Atlantichella calvescens*. Exclusive features or combinations of features of *R. kiwi* are shown in bold. For a more detailed examination of the morphology of *R. bellii*, see Matanov et al. [10], and for *Atlantichella*, Draper et al. [8].

	*Rehubryum bellii*	*Rehubryum kiwi*	*Atlantichella calvescens*
Capsule shape when dry	Short cylindrical to obovoid, not strongly furrowed	Short cylindrical to obovoid, not strongly furrowed	Cylindrical, constricted below mouth, and strongly furrowed
Exothecial bands	Mostly 4 cell rows, reaching the capsule mouth, almost concolor with the exothecial cells	Mostly 2 cell rows, reaching 3–4 rows of suboral cells, darker than the exothecial cells	Mostly 4 cell rows, reaching the capsule mouth, darker than the exothecial cells
Operculum basal rim	Orange to red, not undulate	Crimson, broad, not undulate	Undifferentiated or orangish, undulate
Exostome	Eight pairs of teeth, almost completely divided into sixteen in mature capsules	Eight pairs of teeth remaining unsplit	Eight pairs of teeth, with tendency to completely split into sixteen
Endostome segments	Sixteen, filiform and fragile, shorter than the exostome teeth	Eight, filiform and fragile, shorter than the exostome teeth	Eight, filiform with widened base, as long as the exostome teeth
Endostome connecting membrane	High and protruding mouth	Low, hardly noticeable	Low, frequently incomplete, hardly noticeable

## Data Availability

The sequences generated in this study are available in SRA (https://www.ncbi.nlm.nih.gov/sra/, accessed on 24 July 2025) of NCBI, with the accession number PRJNA1295876.

## Supplementary Materials

The following supporting information can be downloaded at https://www.mdpi.com/article/10.3390/plants14152373/s1: Table S1: Loci summary statistics for the supermatrix; Table S2: Sequences excluded; Table S3: Newly sequenced specimens and selected specimens from Ref. [9].
